# Apoptosis, cytokine and chemokine induction by non-structural 1 (NS1) proteins encoded by different influenza subtypes

**DOI:** 10.1186/1743-422X-8-554

**Published:** 2011-12-21

**Authors:** WY Lam, Apple CM Yeung, Paul KS Chan

**Affiliations:** 1 Department of Microbiology, Faculty of Medicine. The Chinese University of Hong Kong, 1/F Clinical Sciences Building, Prince of Wales Hospital, Shatin, New Territories, Hong Kong Special Administration Region of the People's Republic of China; 2 Stanley Ho Centre for Emerging Infectious Diseases, Faculty of Medicine, The Chinese University of Hong Kong, New Territories, Hong Kong Special Administration Region of the People's Republic of China

**Keywords:** Pandemic influenza, Avian influenza, NS1, Inflammation, Hypercytokinemia, Apoptosis, Pathogenesis

## Abstract

**Background:**

Influenza pandemic remains a serious threat to human health. Viruses of avian origin, H5N1, H7N7 and H9N2, have repeatedly crossed the species barrier to infect humans. Recently, a novel strain originated from swine has evolved to a pandemic. This study aims at improving our understanding on the pathogenic mechanism of influenza viruses, in particular the role of non-structural (NS1) protein in inducing pro-inflammatory and apoptotic responses.

**Methods:**

Human lung epithelial cells (NCI-H292) was used as an *in-vitro *model to study cytokine/chemokine production and apoptosis induced by transfection of NS1 mRNA encoded by seven infleunza subtypes (seasonal and pandemic H1, H2, H3, H5, H7, and H9), respectively.

**Results:**

The results showed that CXCL-10/IP10 was most prominently induced (> 1000 folds) and IL-6 was slightly induced (< 10 folds) by all subtypes. A subtype-dependent pattern was observed for CCL-2/MCP-1, CCL3/MIP-1α, CCL-5/RANTES and CXCL-9/MIG; where induction by H5N1 was much higher than all other subtypes examined. All subtypes induced a similar temporal profile of apoptosis following transfection. The level of apoptosis induced by H5N1 was remarkably higher than all others. The cytokine/chemokine and apoptosis inducing ability of the 2009 pandemic H1N1 was similar to previous seasonal strains.

**Conclusions:**

In conclusion, the NS1 protein encoded by H5N1 carries a remarkably different property as compared to other avian and human subtypes, and is one of the keys to its high pathogenicity. NCI-H292 cells system proves to be a good *in-vitro *model to delineate the property of NS1 proteins.

## Background

Influenza A viruses are major animal and human pathogens with potential to cause pandemics. Avian subtypes H5N1, H7N7 and H9N2 have repeatedly crossed the species barrier to infect humans [1-8]. Since 2003, there have been repeated outbreaks of H5N1 in poultries and sporadic human infections associated with high mortality [8,9]. The recently emerged swine-origin influenza A virus (2009 pandemic H1N1 influenza virus - 2009 pdmH1N1) has spread globally within a few months following the initial detection in Mexico and United States in April 2009, resulting in another influenza pandemic as declared by the World Health Organization (WHO) on June 11 2009 [10]. Although most of the infections are associated with a mild, self-limiting influenza-like illness; the fact that some severe and even fatal outcomes have been observed in individuals without underlying medical conditions poses concerns regarding the pathogenesis of 2009 pdmH1N1 [11,12].

Previous data on human infection with avian influenza virus indicate that cytokine storm is a key mediator, as well as a predictor, for adverse clinical outcomes; especially the haemophagocytic syndrome commonly seen in severe human influenza A H5N1 infections [4,13-16] The preferential infection of deeper lung cells and the prompt induction of apoptosis may also explain the rapid deterioration in lung function [17]. In short, influenza infection can go through a direct pathogenic pathway by inducing apoptosis, and hence cell death and loss of critical function; and alternatively or most probably at the same time through an indirect pathogenic pathway by inducing excessive cytokine/chemokine production from the infected cells. The state of hypercytokinaemia will then trigger adverse consequences such as haemophagocytic syndrome [18].

The virulence of influenza A virus is a polygenic trait. Multiple molecular interactions are involved in determining the outcome of an influenza infection in certain host species [19-28]. The genome of influenza virus is segmented, consisting of eight single-stranded, negative sense RNA molecules, which encode eleven proteins [29]. These are polymerase basic protein 1 (PB1), PB1-F2 protein, polymerase basic protein 2 (PB2), polymerase acidic protein (PA), hemagglutinin (HA), nucleoprotein (NP), neuraminidase (NA), matrix protein 1 (M1), matrix protein 2 (M2), non-structural protein 1 (NS1) and non-structural protein 2 (NS2) [17]. This study focused on NS1 protein which carries multiple functions including the control of temporal synthesis of viral-specific mRNA and viral genomic RNAs [30,31], and interaction with the cellular protein phosphatidylinositol-3-kinase (PI3-kinase) [32-34]; which may cause a delay in virus-induced apoptosis [35]. NS1 protein also has an ability to circumvent the host cell antiviral responses by blocking the activation of RNaseL [36], limiting the induction of interferon (IFN)-β [37-39], interacting with the cellular protein retinoic acid-inducible gene product I (RIG-I) [40-42], blocking host cell mRNA polyadenylation [43,44], blocking the double-stranded-RNA-activated protein kinase (PKR)-mediated inhibition of protein synthesis [31,45], and interacting with cellular PDZ-binding proteins [46]. Furthermore, it has been shown that NS1 protein prevents the maturation of human primary dendritic cells, thereby limiting host T-cell activation [47].

To improve our understanding on the pathogenic mechanism of the newly emerged pandemic strain as well as for influenza viruses in general, we set on this study to examine the property of NS1 proteins encoded by different influenza virus subtypes.

## Methods

### Virus isolates

This study examined the NS1 proteins encoded by seven strains of influenza A viruses including the newly emerged 2009 pandemic H1N1 (A/Auckland/1/2009) (2009 pdmH1N1), an H2 subtype (A/Asia/57/3) (H2N2), an H5N1 isolated from a fatal case in Thailand (A/Thai/KAN1/2004) (H5N1/2004), an H7N3 isolate (A/Canada/504/2004) (H7N3/2004) which caused conjunctivitis and mild upper respiratory tract infection in humans in Canada, an H9N2 isolate (A/Duck/Hong Kong/Y280/1997) (H9N2/1997) that was closely related to those strains found in human H9N2 infections in Hong Kong, and two previous circulating seasonal influenza strains isolated in 2002 (A/HongKong/CUHK-13003/2002) (H1N1/2002) and 2004 (A/HongKong/CUHK-22910/2004) (H3N2/2004). Stocks of these viruses grown in Mandin-Darby canine kidney (MDCK) cells were used.

### Cell cultures

The bronchial epithelial cell line, NCI-H292, derived from human lung mucoepidermoid carcinoma cells (ATCC, CRL-1848, Rockville, MD, USA), were grown as monolayers in RPMI-1640 medium (Invitrogen, Carlsbad, CA) supplemented with 10% fetal bovine serum (FBS), 100 U/mL penicillin and 100 μg/mL streptomycin (all from Gibco, Life Technology, Rockville, Md., USA) at 37°C in a 5% CO_2 _incubator. NCI-H292 cells were used to study the host cellular response to NS1 proteins.

### In-vitro transcription of NS1 mRNA

Viral NS1 mRNA was prepared from an *in-vitro *transcription system. Total RNA was extracted from virus-infected cell lysates using the TRIzol-total RNA extraction kit (Invitrogen). The extracted RNA was eluted in 30 μL of nuclease-free water, and stored in aliquots at -80°C until used. In order to avoid contamination with genomic DNA, the extracted preparation was treated with DNA-Free DNase (Invitrogen). The quality of extracted RNA preparation was assessed by measuring optical density at 260/280 nm with the NanoDrop ND-1000 spectrophotometer (NanoDrop Technologies, Wilmington, DE). cDNA were reversely transcribed from RNA using the SuperScript™ III reverse transcriptase (Invitrogen). DNA fragments containing the coding region of the NS1 gene were linked to a T7 promoter sequence, with or without "hexa histidine-tag" and a polyA tail was created at the end of the fragment by PCR. The PCR was performed using the Platinum^® ^T*aq *DNA polymerase high fidelity (Invitrogen). The primers used for the amplification are listed in Table 1. *In-vitro *transcription was performed using the mMESSAGE mMachine T7 Ultra kit (Ambion, Austin, TX, USA) with 2 h incubation at 37°C. The mRNA was TURBO DNase treated for 15 min at 37°C. Polyadenylation was also performed. The mRNA products were then purified using the MEGAclear kit (Ambion). The quantity and quality of capped mRNA produced was analyzed by measuring optical density at 260/280 nm with the NanoDrop ND-1000 spectrophotometer (NanoDrop Technologies) and by denaturing gel electrophoresis.

**Table 1:** Primers used for amplification of NS1 genes.

Influenza subtypes	Primer sequences
H1N1/2002	Forward: 5'- GATCCTAATACGACTCACTATAGGGAGGAGCAAAAGCAGGGTGGCAA-3' Reverse: 5'- TTTTTTTTTTTTTTTTTTGTAGAAACAAGGGTGTTTTTTATCATT-3'

2009 pdmH1N1	Forward: 5'- GGATCCTAATACGACTCACTATAGGGAGGAGCAAAAGCAGGGTGACAA-3' Reverse: 5'- TTTTTTTTTTTTTTTTTTGTAGAAACAAGGGTGTTTTTTATCATT-3'

H2N2	Forward: 5'- GGATCCTAATACGACTCACTATAGGGAGGAGCAAAAGCAGGGTGACAA-3' Reverse: 5'- TTTTTTTTTTTTTTTTTTGTAGAAACAAGGGTGTTTTTTATCATT-3'

H3N2/2004	Forward: 5'- GGATCCTAATACGACTCACTATAGGGAGGAGCAAAAGCAGGGTGACAA-3' Reverse: 5'- TTTTTTTTTTTTTTTTTTGTAGAAACAAGGGTGTTTTTTATCATT-3'

H5N1/2004	Forward: 5'- GGATCCTAATACGACTCACTATAGGGAGGAGCAAAAGCAGGGTGACAAAG-3' Reverse: 5'- TTTTTTTTTTTTTTTTTTAGTAGAAACAAGGGTGTTTTTTATCAT-3'

H7N3/2004	Forward: 5'- GGATCCTAATACGACTCACTATAGGGAGGAGCAAAAGCAGGGTGACAA-3' Reverse: 5'- TTTTTTTTTTTTTTTTTTGTAGAAACAAGGGTGTTTTTTATCATT-3'

H9N2/1997	Forward: 5'- GGATCCTAATACGACTCACTATAGGGAGGAGCAAAAGCAGGGTGACAA-3' Reverse: 5'- TTTTTTTTTTTTTTTTTTGTAGAAACAAGGGTGTTTTTTATCATT-3'

### Transient transfection system

Approximately 1 × 10^6 ^NCI-H292 cells were transfected with 4 μg of NS1 mRNA in a six-well plate using Lipofectamine™ 2000 (Invitrogen). Briefly, NS1 mRNA and Lipofectamine™ 2000 were incubated together for 30 min in 500 μL of Opti-MEM I before adding to the cells. The transfection efficiency was calculated from the percentage of fluorescent cells that were observed using florescence microscopy after the transfection of fluorescein isothiocyanate (FITC)-labeled short nucleotide primers in separate controls. The transfection efficiency was about 70%. In another control, the effect of the transfection of synthetic double-stranded RNA polyriboinosinic polyribocytidylic acid [poly(I:C)] (Sigma, St Louis, MO), on cytokines/chemokines and apoptosis induction were also observed and took into account for the net effect of mRNA transfection of the experimental cells. The experimentally transfected cells were then collected at 3, 6, 18 and 24 h post-transfection; washed with phosphate-buffered saline (PBS) and trypsinized. After harvesting by centrifugation, the cells were resuspended in a small volume (200-400 μL) of PBS for propidium iodide (PI) staining and FITC-conjugated annexin V (Annexin V-FITC) staining of apoptotic cells.

### Western blot analysis

The mRNA-transfected or non-transfected cell monolayers were lysed, and the total protein concentration was determined by the bicinchoninic acid assay (BCA) (Sigma, St. Louis, MO). Proteins with equivalent concentration were heated for 5 min at 100°C in sample buffer containing β-mercaptoethanol, and were then resolved by 12% or 15% SDS-PAGE. The resolved proteins were transferred to PVDF membrane (Bio-Rad, Richmond, CA) and blocked with 1% powdered milk in Tris-buffered saline with 0.1% Tween 20 (Amersham Pharmacia, Uppsala, Sweden) for 1 h at room temperature. Mouse or rabbit antibodies were then used to probe for His-tagged NS1 (Invitrogen), with overnight incubation at 4°C. The membrane was subsequently incubated for 1 h at room temperature with 1:1000 anti-mouse IgG horseradish peroxidase-linked whole secondary antibody (Amersham Pharmacia, Uppsala, Sweden). The membrane was also probed for GAPDH as a loading control (Chemicon, Temecula, CA).

### Quantification of cytokine/chemokine protein expression using cytometric bead array (CBA)

Cell culture supernatant was collected at 0, 3, 6, 18 and 24 h post-transfection for cytokine/chemokine measurement by the Cytometric Bead Array (CBA) Soluble Protein Flex Set system (BD Biosciences, San Jose, CA) using the BD FACSAria Flow Cytometer System (BD Biosciences) according to the manufacturer's instructions. Six cytokines/chemokines (CCL-2/MCP-1, CCL-3/MIP-1α, CCL-5/RANTES, CXCL-9/MIG, CXCL-10/IP-10, IL-6) were measured. The results were expressed as number of fold changes with reference to the levels of non-transfected cell controls. All experiments were performed in triplicates.

### Propidium iodide (PI) staining and DNA content analysis by flow cytometry

The overall (early and late phases together) proportion of apoptotic cells was measured by PI flow cytometric assay which is based on the principle that apoptotic cells are characterized by DNA fragmentation and the consequent loss of nuclear DNA content at the late phase of apoptosis. Briefly, transfected cells (10^6^) were washed with PBS and fixed with 70% ethanol overnight at 4°C. The fixed cells were then stained with 50 μg/mL of PI (Sigma, St. Louis, MO) with 1 μg of RNase A/mL at 4°C for 1 h. PI binds to DNA by intercalating between the bases, with no sequence preference. The DNA contents of the cells were then analyzed by flow cytometry (FACSAria; BD Biosciences, San Jose, CA). Cells at the late phase of apoptosis, i.e., sub-G1 (hypodiploid) cells, will have DNA contents lower than those of G1 cells. The proportions of these apoptotic cells, i.e., sub-G1 cells, at different time points post-transfection were determined. Staurosporine-treated cells were also used as a positive control. All experiments were performed in triplicates.

### Annexin V-FITC staining of apoptotic cells and analysis by flow cytometry

Annexin V-FITC staining was used to measure the proportion of cells that were at the early phase of apoptosis. Cells were collected, washed with PBS, and stained with FITC-conjugated annexin V (BD Biosciences, Franklin Lakes, NJ) and PI for 20 min at room temperature in dark. The stained cells were then analyzed by flow cytometry (FACSAria; BD Biosciences). FITC-conjugated annexin V binds to surface phosphatidylserine translocated from the intra- to the extracellular plasma membrane early in apoptosis. Cells were simultaneously stained with PI to discriminate membrane-permeable necrotic cells from FITC-labeled apoptotic cells. Apoptotic cells were identified as those with annexin V-FITC staining only, and the results were expressed as the proportion of these cells among the total number of cells analyzed. Staurosporine-treated cells were also used as a positive control. All experiments were performed in triplicates.

## Results

### Cytokine/chemokine expression profiles

**Figure 1:**
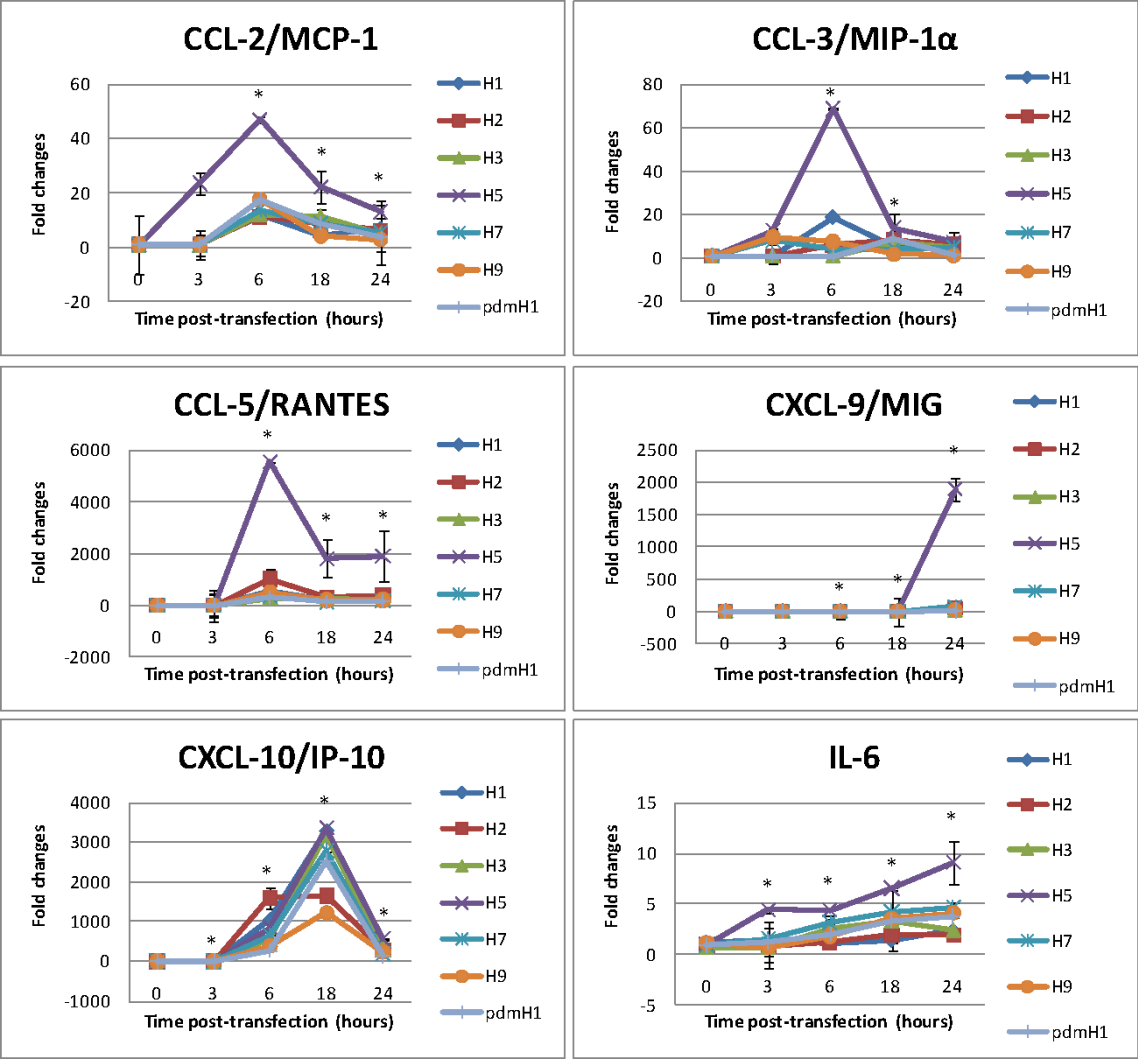
**Cytokines/chemokines induced by transfection of NS1 mRNA of different influenza subtypes**. NCI-H292 cells were transfected with NS1 mRNA derived from seven subtypes of influenza A including seasonal H1N1 (H1), H2N2, H3N2, H5N1, H7N3, H9N2 and 2009 pandemic H1N1 (pdmH1). Cytokine/chemokine levels are expressed as fold-changes compared to the non-transfected cell controls ± SD (p* < 0.05) measured at the same time points.

Different profiles of cytokine/chemokine expression were observed following the transfection of NS1 mRNA derived from various influenza subtypes (Figure 1). Overall, the most prominent induction was observed for CXCL-10/IP-10 regardless of influenza subtype, and with > 1,000-fold increase at peak induction. IL-6 was also induced by all subtypes but to a much lower extent (< 10-fold increase at peak induction). A distinct pattern in induction between H5 and other subtypes was observed for CCL-2/MCP-1, CCL-3/MIP-1α, CCL-5/RANTES and CXCL-9/MIG. For all these four cytokines/chemokines, the level of expression induced by H5 was much higher than other subtypes.

The six studied cytokines/chemokines can be divided in to two groups according to the time to reach peak level of induction. The "early" group included CCL-2/MCP-1, CCL-3/MIP-1α and CCL-5/RANTES showing a peak at 6 h post-transfection. All members of the "early" group showed a strong response to H5, but they were only induced to low levels by other subtypes (Figure 1). The "late" group included IL-6, CXCL-9/MIG and CXCL-10/IP-10 showing a peak at 18-24 h post-transfection. No obvious correlation between the time to reach peak level and the subtype of virus was observed.

Among the seven subtypes of influenza examined, only H5 showed a distinct profile of cytokine/chemokine induction; and being the strongest inducer for all the six cytokines/chemokines. The cytokine/chemokine induction profiles observed for the recently emerged 2009 pdmH1N1-NS1 were similar to previous seasonal strains.

### Apoptosis

**Figure 2:**
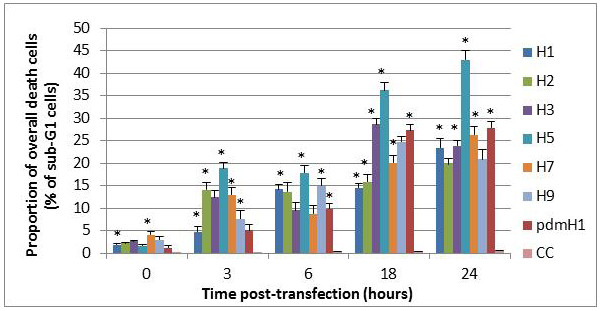
**Proportion of overall apoptotic cells induced by transfection of NS1 mRNA of different influenza subtypes**. NCI-H292 cells were transfected with NS1 mRNA derived from seven subtypes of influenza A including seasonal H1N1 (H1), H2N2, H3N2, H5N1, H7N3, H9N2 and 2009 pandemic H1N1 (pdmH1). CC represents non-transfected controls. The overall proportion of death cells was measured by propidium iodide staining using flow cytometry with the sub-G1 cell proportion counted ± SD (p* < 0.05).

The overall (early and late phases together) proportion of apoptotic cells for H5N1/2004-NS1 was the highest at all time points as compared to other subtypes (Figure 2). At 24 h post-transfection, 44% of H5N1-NS1-transfected cells had entered apoptosis, compared to 20-28% of other subtypes. The 2009 pdmH1N1-, H3N2-, and H9N2-transfected cells showed a relatively higher proportion of apoptosis (25-29%) at 18 h post-transfection; however these subtypes reached similar levels as compared to other non-H5 subtypes at 24 h post-transfection (Figure 2).

**Figure 3:**
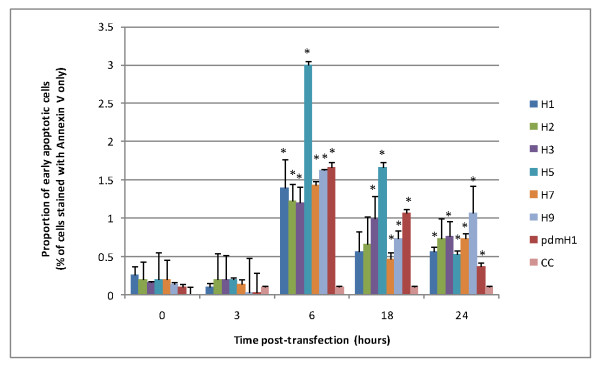
**Proportion of early apoptotic cells induced by transfection of NS1 mRNA of different influenza subtypes**. NCI-H292 cells were transfected with NS1 mRNA derived from seven subtypes of influenza A including seasonal H1N1 (H1), H2N2, H3N2, H5N1, H7N3, H9N2 and 2009 pandemic H1N1 (pdmH1). CC represents non-transfected controls. Cells at early apoptotic phase as stained positive for annexin V but not propidium iodide were identified by flow cytometry ± SD (p* < 0.05).

Figure 3 shows the proportion of cells at the early phase of apoptosis at different time points post-transfection. Overall, apoptotic activity was the highest at 6 h post-transfection, and then decreased gradually. This time course of apoptosis was similar among the different subtypes of influenza viruses examined. Figure 3 displays the distinct apoptotic induction ability of H5N1 as compared to other subtypes. At the peak of apoptotic activity (6 h post-transfection), the proportion of apoptotic cells for H5N1 was about twice higher than those for other subtypes. The newly emerged 2009 pdmH1N1-NS1 showed a moderate ability in inducing apoptosis. At both 6 h and 18 h post-transfection, the proportion of early apoptotic cells ranked second, just following H5N1/2004-NS1, among the subtypes examined.

## Discussion

In this study, the pathogenic properties of NS1 proteins encoded by different subtypes of influenza A viruses were compared by measuring cytokine/chemokine expression and apoptosis induced in transfected cells.

While the key target of influenza virus is lung epithelial cells [48,49], most studies on influenza pathogenesis have been based on macrophages and monocytes infected *in-vitro *or *in-vivo *[50-52]. It is important to note that at the time of early infection these immune cells would not be present in great numbers until they have been recruited into the area. The mechanism concerning bronchial infiltration of inflammatory cells, particularly lymphocytes and eosinophils, and the subsequent hyperresponsiveness of the bronchial wall induced by viruses remain unclear [53]. Therefore, in this study we have used a cell line derived from human lung epithelial cells as an *in-vitro *model to study the pathogenicity of influenza NS1 proteins.

Previous *in-vitro *studies have shown that influenza infection induces the production of cytokines IFN-α, tumor necrosis factor (TNF)-α, IL-1, IL-6, IL-8 and the mononuclear cell attractant chemokines CCL-3/MIP-1α, CCL-4/MIP-1β, CCL-2/MCP-1, CCL-7/MCP-3, CXCL-10/IP-10 and CCL-5/RANTES in human monocytes, epithelial cells, rat alveolar or murine macrophages [48,50,53-62]. Based on the findings of these studies, we identified the six key cytokines/chemokines for the current study.

Recently, it has been shown that the inflammatory response is played out over time in a reproducible and organized way with different induction kinetics after an initiating stimulus [63]. Cytokines released following infection can be classified broadly into "early" and "late" cytokines. Our results showed that CCL-2/MCP-1, CCL-3/MIP-1α and CCL-5/RANTES were produced early post-transfection; while IL-6, CXCL-10/IP-10 and CXCL-9/MIG were produced later. This time course of cytokine/chemokine production was consistently observed across different subtypes of influenza viruses with different pathogenicity. It would be worthwhile to further investigate whether this temporal sequence is unique to influenza or generally true for other acute respiratory viruses.

The most remarkable observation in this study was the distinct cytokine/chemokine profiles induced by the NS1 protein of H5N1. Our *in-vitro *observation is in line with previous reports that the peripheral blood of patients infected with H5N1 have much higher serum levels of CXCL-10/IP-10 and CCL-2/MCP-1 than patients infected with seasonal influenza [13,15]. Furthermore, the *in-vitro *model used in our study by measuring the levels of cytokines in lung tissue may be more relevant to pathogenesis than levels in blood [15]. Another *in-vitro *study in macrophages also showed a stronger cytokine induction by H5N1/1997 viruses compared to H3N2 [51].

We found that NS1 protein encoded by 2009 pdmH1N1 virus induced similar levels of cytokine/chemokine compared to seasonal H1N1 and H3N2 strains. This observation is in line with a recent report which showed that pro-inflammatory cytokine expression in the 2009 pandemic H1N1 virus-infected macrophages was similar to that of seasonal H1N1/1999, and was much lower than in H5N1/2004-infected cells [64-67].

In this study, we observed a similar temporal profile of apoptosis as induced by different subtypes of influenza. This is in contrast to previous studies based on whole virions. For instance, Geiler et al. (2011) [67] reported a delay in the induction of apoptosis for 2009 pdmH1N1 compared to H5N1; whereas Mok et al. (2007) [68] reported a delayed apoptosis of H5N1 compared to seasonal H1N1. The reason for these different observations remains to be verified. Both of these two studies [67,68] used whole virions, and therefore the observation may be partly related to the time required for sufficient virus replication and hence the synthesis of a certain amount of NS1 protein; whereas the current study used transfection where the same amount of NS1 was expected to be synthesized at any one time for different subtypes. If NS1 protein *per se *was the question of interest, using transfection methods with the same transfection efficiencies among different subtypes, might avoid biases result from differences in replication efficiency of the viruses being studied. Another major difference is that in contrast to the lung epithelial cells used in our study, the two previous studies used macrophages. These two cell types may display differences in apoptotic response to different subtypes of influenza.

Another remarkable observation of the current study is the high apoptosis inducing ability conferred by NS1 protein encoded by H5N1 compared to all other subtypes. This is reminiscent of the rapid development of severe primary pneumonitis in patients infected with H5N1 [4,13-16]. Our data showed that the apoptosis inducing ability of NS1 protein encoded by 2009 pdmH1N1 virus was similar to H7, H9 and seasonal subtypes; but much lower than H5N1. This is in line with a previous observation based on macrophages infected with whole virions, where the level of apoptosis induced by 2009 pdmH1N1 was much lower than H5N1 [67].

NS1 have both pro- and anti-apoptotic functions, and the level of apoptosis observed reflects a balance between the two [30,34,69-73]. It would be worthwhile to further investigate whether the NS1 encoded by H5N1 is more pro-apoptotic or less anti-apoptotic as compared to other subtypes.

## Conclusions

This study confirms that transfection of human lung epithelial cell line with NS1 mRNA is a suitable *in-vitro *model to delineate and compare the function of NS1 proteins encoded by different influenza subtypes. Our data indicates that NS1 protein is one of the keys for an exceptional higher pathogenicity of H5N1 compared to other avian and human subtypes. The NS1 protein encoded by 2009 pdmH1N1 exhibits a similar pro-inflammatory and apoptosis inducing ability as with other human seasonal subtypes, reflecting their similarity in pathogenicity. It is worthwhile to further explore the potential of using NS1 protein as a target of therapeutic intervention.

## Abbreviations

2009 pdmH1N1: 2009 pandemic H1N1 influenza virus; Annexin V-FITC: fluorescein isothiocyanate-conjugated annexin V staining; BCA: bicinchoninic acid assay; CBA: Cytometric Bead Array; CCL-2/MCP-1: Chemokine (C-C motif) ligand 2/monocyte chemotactic protein-1; CCL-3/MIP-1α: Chemokine (C-C motif) ligand 3/Macrophage inflammatory protein-1α; CCL-4/MIP-1β: Chemokine (C-C motif) ligand 3/Macrophage inflammatory protein-1β; CCL-5/RANTES: Chemokine (C-C motif) ligand 5/Regulated upon Activation, Normal T-cell Expressed, and Secreted; CCL-7/MCP-3: Chemokine (C-C motif) ligand 2/monocyte chemotactic protein-3; cDNA: complementary Deoxyribonucleic Acid; CXCL-9/MIG: Chemokine (C-X-C motif) ligand 9/Monokine induced by gamma interferon; CXCL-10/IP-10: C-X-C motif chemokine 10/Interferon gamma-induced protein 10; FBS: fetal bovine serum; FITC: fluorescein isothiocyanate; GAPDH: Glyceraldehyde 3-phosphate dehydrogenase; HA: hemagglutinin; H1N1/2002: (A/HongKong/CUHK-13003/2002); H2N2: H2 subtype (A/Asia/57/3); H3N2/2004: (A/HongKong/CUHK-22910/2004); H5N1/2004: (A/Thai/KAN1/2004); H7N3/2004: (A/Canada/504/2004); H9N2: (A/Duck/Hong Kong/Y280/1997); IFN-α: Interferon alpha; IFN-β: Interferon beta; IgG: immunoglobulin G; IL-1: Interleukin 1; IL-6: Interleukin 6; IL-8: Interleukin 8; M1: matrix protein 1; M2: matrix protein 2; MDCK cells: Mandin-Darby canine kidney cells; mRNA: messenger Ribonucleic Acid; NA: neuraminidase; NP: nucleoprotein; NS1: non-structural protein 1; NS2: non-structural protein 2; PA: polymerase acidic protein; PB1: polymerase basic protein 1; PB2: polymerase basic protein 2; PBS: phosphate-buffered saline; PI staining: propidium iodide staining; PI3-kinase: phosphatidylinositol-3-kinase; PKR: double-stranded-RNA-activated protein kinase; poly(I:C): polyriboinosinic polyribocytidylic acid; PVDF membrane: Polyvinylidene fluoride membrane; RIG-I: retinoic acid-inducible gene product I; RNaseL: 2-5A-dependent ribonuclease; RPMI-1640 medium: Roswell Park Memorial Institute 1640 medium; SDS-PAGE: sodium dodecyl sulfate polyacrylamide gel electrophoresis; TNF-α: tumor necrosis factor-α; WHO: World Health Organization

## Competing interests

The authors declare that they have no competing interests.

## Authors' contributions

ACMY performed the *in-vitro *transcription assays, RT-PCR assays, apoptotic cells staining and flow-cytometry assays. WYL was responsible for experimental design, analyses and drafting of the manuscript. PKSC was responsible for design and supervision of the study. All authors read and approved the final manuscript.
